# Top-Down Mass Spectrometry and Its Current Applications in Biomarker Discovery in Aging and Age-Related Diseases

**DOI:** 10.3390/ijms27083610

**Published:** 2026-04-18

**Authors:** Eun Ju Lee, Haneul Choi, Ki Ha Min, Hae-Min Park, Seung Pil Pack

**Affiliations:** 1 Department of Chemical Engineering and Applied Chemistry, Chungnam National University, Daejeon 34134, Republic of Korea; exnju1005@o.cnu.ac.kr (E.J.L.); chn8846@o.cnu.ac.kr (H.C.); 2 Department of Biotechnology and Bioinformatics, Korea University, Sejong 30019, Republic of Korea; alsrlgk@korea.ac.kr

**Keywords:** top-down mass spectrometry, aging, age-related diseases, proteoform biomarkers

## Abstract

Aging is one of the most complex biological processes, which leads to a gradual decline in the function of organs, tissues and cells, and significant increases in the risks of many age-associated diseases, including cancer, neurodegenerative disorders, and cardiovascular diseases. Protein biomarkers have attracted increasing attention in research on aging and age-related diseases. Considering the fact that proteins are large heterogenous biomolecules due to coding polymorphisms, alternative RNA splicing and post-translational modifications (PTMs), including glycosylation, phosphorylation, and methylation, mass spectrometry (MS)-based top-down proteomics (TDP) is a powerful technology that allows for measuring proteins without proteolysis, thus characterizing intact forms of proteins, which provides information on primary sequences, including their modifications. This review provides an overview of TDP technologies, with a particular focus on the separation, ionization, and fragmentation of intact proteins and introduces the most recent applications of TDP to the discovery of proteoform-resolved biomarkers associated with aging and age-related diseases.

## 1. Introduction

Mass spectrometry (MS) is a powerful technology for characterizing protein primary structures, including their modifications. Two major MS-based approaches, the bottom-up and top-down approaches, have been most widely used to identify and characterize whole proteins of complex mixtures [1]. In the traditional bottom-up workflow, intact proteins are enzymatically digested into smaller peptides [2,3]. The resulting peptide mixtures are separated by liquid chromatography (LC) and introduced into a mass spectrometer, where peptide ions are subjected to tandem MS analysis using either collision-induced dissociation (CID) or higher-energy collisional dissociation (HCD) to generate fragment ion spectra [3,4]. Proteins are then inferred computationally by matching experimental tandem MS spectra to theoretical fragmentation patterns obtained from protein sequence databases. Over the past two decades, the bottom-up approach has enabled the comprehensive analysis of proteomes from diverse and complex biological systems, including cells, tissues, and organisms, providing insights into protein expression, signaling networks, and disease mechanisms. The main advantage of bottom-up proteomics lies in its high throughput, dynamic range, and sensitivity, allowing identification and quantification of thousands of proteins from complex mixtures through efficient peptide separation and high ionization efficiency [5,6,7]. Furthermore, recent advances in bottom-up proteomics (BUP), including data-independent acquisition and label-free quantification, have improved reproducibility and quantitative precision and accuracy, expanding the scope of large-scale protein identification and quantification [8,9]. In addition, universal solid-phase protein preparation (USP3) has recently improved BUP sample processing by enabling efficient protein capture, cleanup, and digestion using paramagnetic beads [10]. This approach could minimize sample loss and enhance reproducibility, particularly for low-input or heterogeneous clinical specimens commonly observed in aging studies. However, the bottom-up approach has intrinsic limitations related to enzymatic digestion. Trypsin digestion produces peptides that are often too short for MS detection and are therefore frequently not identified [11,12]. Additionally, peptide-based analysis inherently prevents direct measurement of alternative splicing, post-translational modifications (PTMs), and combinatorial modifications within intact proteins [11,13].

To overcome the intrinsic limitations of peptide-centric analyses, the top-down approach has emerged as a powerful complementary MS technology. In contrast to the BUP, top-down proteomics (TDP) analyzes intact proteins directly without prior enzymatic digestion, thereby preserving their covalent and non-covalent structures [14,15]. In this top-down approach, intact proteins are separated by high-performance chromatographic or electrophoretic methods such as reversed-phase liquid chromatography (RP-HPLC), size-exclusion chromatography (SEC), or capillary electrophoresis (CE), and subsequently introduced into a high-resolution mass spectrometer. The resulting intact protein ions are isolated and fragmented within the mass spectrometer using fragmentation techniques such as collision-induced dissociation (CID), electron capture dissociation (ECD), or ultraviolet photodissociation (UVPD), generating fragment ion spectra that directly characterize the primary sequence and PTMs of proteins [16]. Compared to BUP, the major advantage of the TDP is its ability to provide proteoform-level resolution, enabling the comprehensive characterization of all molecular variants arising from genetic variation, alternative splicing, and PTMs within a single protein species. This direct measurement offers a solution to the protein inference problem intrinsic to bottom-up strategies and provides better understanding of proteomic heterogeneity and protein isoform function.

In addition to genetic variation, alternative splicing, and PTMs within a single protein, protein assemblies, including protein–protein and protein–ligand interactions, play critical roles in cellular signaling [17]. TDP approaches have proven successful in identifying a large number of proteins present in complex biological systems, including cells, tissues, and organs [18,19,20]. The majority of these approaches have been performed under denaturing conditions, due to the improvements in fragmentation efficiency. While denaturing TDP preserves covalently linked post-translationally modified forms of proteins present in vivo and enables detailed proteoform-resolved characterization, it is intrinsically limited because it inherently disrupts noncovalent protein–protein and protein–ligand interactions. In contrast, native TDP offers the advantage of enabling the analysis of intact protein complexes ranging in size from approximately 30 kDa to 800 kDa [21,22,23]. Under denaturing conditions, proteins larger than 30 kDa typically display broad charge state distributions, which substantially reduce MS signal intensity. However, these proteins exhibit significantly fewer charge states and a narrower charge state distribution within a lower *m*/*z* range under native conditions, leading to enhanced sensitivity for large protein complexes [24].

**Figure 1:**
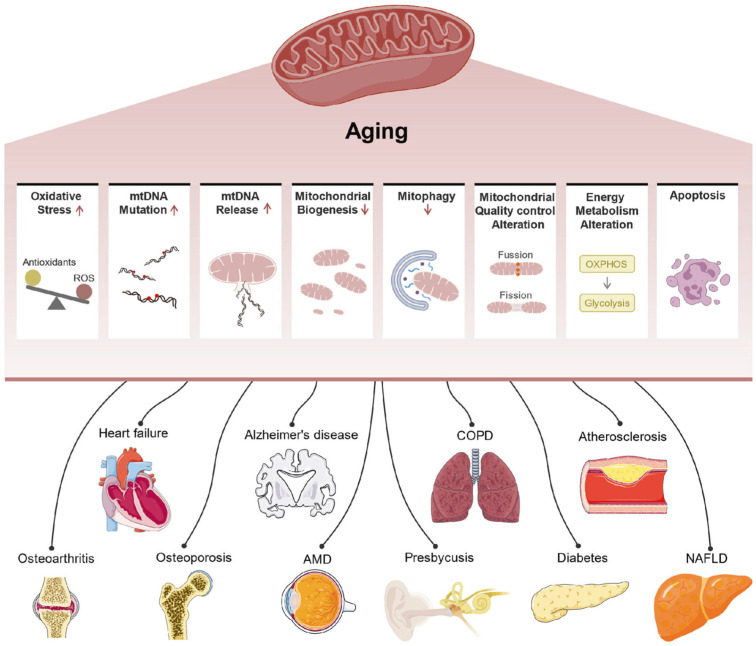
Mitochondrial dysfunction contributes to diverse aging-related diseases (reproduced from Guo et al. [25], Signal Transduction and Targeted Therapy; published by Springer Nature Limited, 2022).

**Figure 2:**
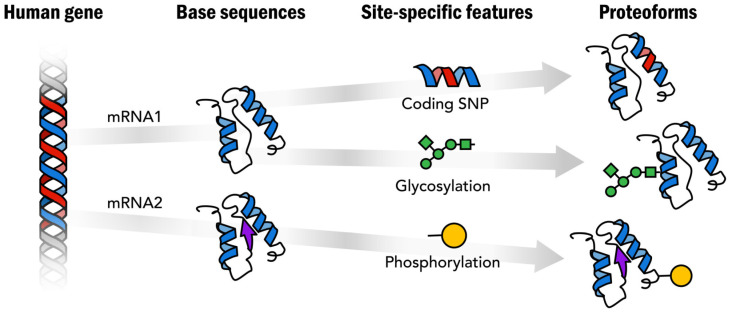
Proteoforms: distinct protein forms arising from a single gene (reproduced from Smith et al. [32], Science Advances; published by American Association for the Advancement of Science, 2021).

**Figure 3:**
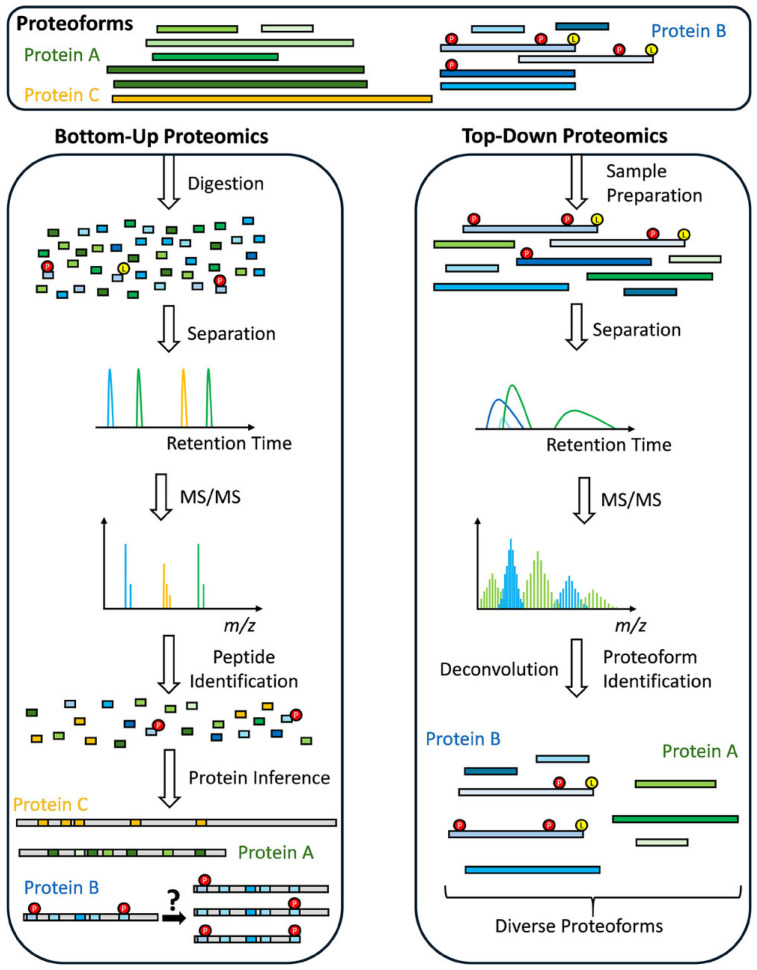
Schematic overview of BUP and TDP (reproduced from Kaulich et al. [33], Proteomics; published by Wiley-VCH, 2025).

Aging is one of the most complex biological processes, which leads to a gradual decline in function of organs, tissues and cells, and significant increases in the risks of many age-associated diseases, including cancer, neurodegenerative disorders, and cardiovascular diseases, related to mitochondrial dysfunction (Figure 1). Protein biomarkers have attracted increasing attention in research on aging and age-related diseases because understanding the molecular mechanisms underlying aging is crucial for identifying interventions and treatments for aging-related diseases [25]. Proteins represent highly heterogeneous biomolecules due to genetic variation, alternative splicing, and combinatorial PTMs including glycosylation, phosphorylation, and methylation (Figure 2). Importantly, aging-associated phenotypes frequently arise from dysregulation of signaling pathways and proteostasis networks rather than large-scale protein abundance changes [26,27]. Peptide-based measurements obtained through BUP often average signals across these heterogeneous molecular species, potentially masking biologically meaningful alterations that occur at the proteoform level. As a result, abundance changes detected by BUP do not necessarily reflect functional protein states, limiting their specificity as aging biomarkers. In contrast, proteoform-level analysis enables the detection of subtle regulatory alterations, including PTM cross-talk and coordinated modification patterns across functional domains, which are central features of cellular aging. Several reviews have summarized the related topics, including sample preparation, quantification, bioinformatics tools and applications in basic and clinical research [28,29,30,31]. In this review, we offer an overview of TDP technologies (Figure 3), with a particular focus on the separation, ionization, and fragmentation of intact proteins. This review also focuses on recent studies reflecting their applications in aging and age-related diseases.

## 2. Top-Down Mass Spectrometry Techniques

### 2.1. Intact Protein Separation

Liquid chromatography (LC) remains a central separation platform in top-down proteomics (TDP). However, recent developments have shifted the focus from conventional chromatographic principles toward improving proteoform preservation, MS compatibility, and proteome coverage. Rather than serving merely as a generic separation tool, modern LC strategies are increasingly engineered to address challenges unique to intact protein analysis, including limited recovery of large proteoforms, on-column denaturation, and insufficient resolution for highly heterogeneous proteoform populations [28,29]. Among LC modalities, reversed-phase liquid chromatography (RPLC) continues to be widely implemented in TDP, but recent advances primarily involve innovations in stationary phase design and separation conditions optimized for intact proteins. Short-chain alkyl stationary phases (e.g., C4 and C5) have improved protein recovery by reducing irreversible adsorption [34,35], while superficially porous and polymer-based materials have enhanced mass transfer and peak capacity for large proteoforms [16,35,36,37]. The development of wide-pore columns (≥300 Å) has further enabled the efficient analysis of high-molecular-weight species such as antibodies [38,39,40]. More recently, methodological efforts have focused on mitigating denaturation effects associated with conventional RPLC-MS conditions [41,42]. Strategies including reduced trifluoroacetic acid concentrations, lower column temperatures, and alternative volatile modifiers have been introduced to preserve higher-order protein structures while maintaining ionization efficiency and MS sensitivity [42]. These refinements are particularly important for extending RPLC applications toward native or near-native TDP workflows. Size-exclusion chromatography (SEC) has evolved from a traditional prefractionation tool into an MS-compatible platform through the adoption of volatile buffer systems such as ammonium acetate and ammonium formate [42]. These developments have enabled direct SEC-MS coupling and facilitated the analysis of challenging analytes, including membrane proteins and intact protein complexes. Recent studies have also examined protein–stationary phase interactions during SEC separations, highlighting their influence on structural integrity and proteoform stability [43]. Despite inherently limited chromatographic resolution, SEC is increasingly integrated into multidimensional workflows where its orthogonality to RPLC enhances overall proteoform coverage. Ion-exchange chromatography (IEX) has similarly undergone methodological refinement aimed at improving MS compatibility. The replacement of nonvolatile salt gradients with volatile buffering systems has enabled direct IEX-MS coupling, reducing sample handling and minimizing proteoform loss during desalting [44,45,46]. Recent implementations emphasize charge-based prefractionation strategies that expand detectable proteoform diversity when combined with downstream high-resolution MS analysis, positioning IEX as a complementary dimension within multidimensional TDP pipelines rather than a standalone separation method. More recently, the Zhu group introduced a platform that incorporates the microfluidic sample preparation system nanoPOTS into an LC-MS-based TDP workflow, enabling sensitive proteoform analysis from limited mammalian cell inputs [47].

In parallel with LC developments, capillary electrophoresis (CE) has emerged as a highly impactful separation technology for TDP owing to its ultralow flow rates and exceptional separation efficiency [48,49,50]. Recent innovations have focused on improving sensitivity, automation, and multidimensional integration rather than fundamental electrophoretic principles. Advances such as spray-capillary interfaces have enabled picogram-level proteoform analysis, demonstrating the feasibility of TDP from extremely limited biological samples [51]. Multidimensional platforms integrating SEC- or RPLC-based prefractionation with capillary zone electrophoresis (CZE)-MS have substantially expanded proteome coverage, achieving large-scale proteoform identification in microbial systems [52]. In addition, automated capillary isoelectric focusing (cIEF)-MS workflows have enabled high-resolution and higher-throughput proteoform profiling, including rapid analyses of complex protein corona samples [53,54]. Despite these advances, CE-based TDP still faces challenges related to protein adsorption to capillary surfaces and long-term reproducibility [55]. Current research therefore focuses on improved capillary coatings, interface stability, and standardized workflows to enhance robustness for broader biological and clinical applications [48].

Furthermore, multidimensional separation techniques, which is the combination of two or more separation methods with high orthogonality, have been recently developed and applied that demonstrate improved separation resolution and more comprehensive identification of intact proteins in TDP [56]. For RPLC-based multidimensional separation, the use of high-pH/low-pH RPLC platforms achieved good orthogonality, improved separation efficiency, and more comprehensive identification of proteoforms compared with one-dimensional RPLC for TDP [57]. Most recently, the Tholey group have developed a two-dimensional low pH/low pH RPLC separation method that uses different ion pairing reagents and stationary phases between the two RPLC dimensions, which enables the identification a higher number of proteoforms smaller than 20 kDa, compared to the high-pH/low-pH approach [58]. For SEC-based multidimensional separation, the Ge group introduced a serial size exclusion chromatography (sSEC) technique to enable high-resolution size-based fractionation of intact proteins up to ~223 kDa from complex protein mixtures. In this study, two-dimensional (2D) sSEC-RPC (reverse-phase chromatography) separation of proteins have enhanced the detection of high molecular weight proteins [59]. Recently, the Wang group has developed a new separation technique called small-scale serial Size Exclusion Chromatography (s3SEC), which incorporates a small-scale protein extraction and serial SEC without post-fractionation sample handling, coupled to online RPLC-MS for intact protein analysis. This technique significantly improved the sensitivity and reduced the proteome complexity across the fractions [60]. For IEX-based multidimensional separation, IEC coupled to RPLC has been commonly used for intact protein separation from cellular complex mixtures such as yeast and human cells [61,62]. In addition, the Tholey group has recently established a sample preparation approach that combines SCX-prefactionation and RPLC-MS, which has enhanced the identification and characterization of short open reading frame (sORF)-encoded peptides in TDP [63]. Most recently, the Li group has introduced a two-dimensional LC–MS platform that coupled high-resolution anion-exchange chromatography (AEX) as the first dimension to RPLC as the second dimension for multi-attribute characterization of adeno-associated viruses (AAVs) [64].

### 2.2. Ionization and Fragmentation of Intact Proteins

Ionization and fragmentation strategies represent critical determinants of analytical depth in TDP, and recent developments have focused less on fundamental ion generation principles and more on improving sensitivity, structural preservation, and sequence coverage for large and heterogeneous proteoforms. Modern TDP workflows increasingly integrate advanced ionization interfaces and hybrid activation methods designed specifically to overcome challenges associated with intact protein analysis. Electrospray ionization (ESI) remains the dominant ionization platform for TDP [65]. However, recent progress has primarily centered on improving compatibility with native conditions, low-abundance samples, and spatially resolved analyses rather than refining its underlying mechanism. Advances in nanoESI and optimized emitter geometries have enhanced ionization efficiency while minimizing sample consumption, enabling the analysis of limited clinical and single-cell-level materials [66]. Furthermore, developments in native ESI workflows have facilitated the preservation of higher-order protein structures and noncovalent complexes, expanding TDP applications toward structural proteomics [23]. Ambient ionization approaches such as desorption electrospray ionization (DESI) have further extended TDP capabilities by enabling direct, preparation-free analysis of intact proteins from biological surfaces, supporting spatially resolved proteoform characterization in tissues [67]. Matrix-assisted laser desorption/ionization (MALDI) has similarly evolved beyond conventional MALDI–TOF implementations through integration with high-resolution mass analyzers, including Orbitrap and Fourier transform ion cyclotron resonance (FTICR) instruments [68]. These developments have substantially improved mass accuracy and proteoform discrimination, addressing historical limitations associated with resolution and fragmentation efficiency. In parallel, coupling MALDI with imaging mass spectrometry (MALDI-IMS) has enabled in situ mapping of intact proteoforms, representing an important advance toward spatial proteomics and clinical tissue analysis [69]. Recent ambient surface sampling and imaging workflows now allow minimally invasive, spatially resolved proteoform detection directly from biological specimens, highlighting an emerging convergence between TDP and molecular histology [68,70].

Technological innovation has been particularly transformative in fragmentation methodologies, where efforts increasingly aim to improve sequence coverage for large proteins while preserving labile post-translational modifications (PTMs). Collision-based activation methods such as collision-induced dissociation (CID) and higher-energy collisional dissociation (HCD) remain widely used but are now primarily employed within hybrid workflows due to their limited efficiency for highly folded or high-mass proteoforms [71,72]. Recent implementations leverage optimized energy deposition and high-resolution Orbitrap detection to enhance fragment ion characterization while maintaining analytical robustness. Electron-based dissociation techniques, including electron capture dissociation (ECD) and electron transfer dissociation (ETD), have undergone significant instrumental expansion beyond their initial platform constraints [73]. Advances such as ECD implementations compatible with Q-TOF instruments and improved ETD reaction control have broadened accessibility and throughput for discovery-driven TDP [74]. These methods remain particularly valuable for preserving labile PTMs and noncovalent interactions, making them central to proteoform-resolved characterization of regulatory protein modifications. Photodissociation-based activation, especially ultraviolet photodissociation (UVPD), represents one of the most impactful recent advances in top-down MS [75]. High-energy photon absorption enables rapid and extensive backbone cleavage, generating diverse fragment ion series and substantially improving sequence coverage for large proteins [76,77]. Integration of UVPD with high-resolution analyzers has enabled interpretation of complex fragmentation patterns [76,78,79,80], while recent hybrid activation strategies, including UVPD–ETD and UVPD–HCD workflows, demonstrate synergistic improvements in proteoform characterization, particularly for large biomolecules such as antibodies [81,82,83]. These hybrid approaches exemplify a broader trend toward multi-modal ion activation designed to overcome the intrinsic limitations of individual fragmentation techniques.

### 2.3. Limitations of TDP

Recently, an increasing number of studies have highlighted the potential of top-down proteomics to elucidate disease mechanisms and identify novel biomarkers through the direct characterization of intact proteoforms [84,85]. Despite these advances, several intrinsic technical challenges continue to limit the broader application of top-down approaches. First, protein purification and separation remain substantially more demanding than peptide-level workflows. The extensive dynamic range of protein expression and the inherent complexity of the proteome necessitate efficient fractionation and separation prior to mass spectrometry MS analysis to achieve comprehensive proteome coverage [86]. Whereas bottom-up proteomics benefits from well-established gel- and LC-based separation strategies, high-throughput, rapid, and MS-compatible methods for intact protein separation remain comparatively underdeveloped [84]. In particular, intact proteins, especially membrane proteins and high-molecular-weight proteoforms, present significant solubility challenges. Surfactants commonly used to enhance protein solubility (e.g., SDS) are frequently incompatible with downstream MS analysis, often resulting in sample loss, reduced reproducibility, and incomplete proteome recovery [87]. Second, detection sensitivity represents a major analytical bottleneck, particularly for low-abundance and large proteoforms. In top-down analyses, signal intensity is distributed across multiple charge states and broad isotopic envelopes, which decreases signal-to-noise ratios and complicates the identification of low-level proteoforms without prior enrichment or advanced fractionation strategies [29]. Third, accurate characterization of intact proteoforms requires mass analyzers with exceptionally high resolving power and mass accuracy. Such performance is necessary to discriminate subtle mass differences arising from post-translational modifications, disulfide bonds, and proteoform variants that may differ by only a few Daltons or less [29]. Consequently, top-down workflows predominantly rely on Fourier transform ion cyclotron resonance (FT-ICR) or Orbitrap mass spectrometers, as lower-resolution instruments generally lack sufficient resolving capability for reliable intact proteoform analysis [29,88]. Taken together, these limitations highlight the need for continued methodological innovation in intact protein separation, sensitivity enhancement, and high-resolution mass spectrometry. Advances in these areas are expected to substantially expand the analytical depth and practical utility of top-down proteomics, ultimately enabling more comprehensive insights into protein function and disease biology.

Despite progress in LC- or CE-based separation techniques and MS instrumentation, several practical challenges still limit the clinical translation of TDP. In particular, the issues related to reproducibility, analytical standardization and sample throughput remain major limitations to the application of proteoform-resolved measurements in clinical studies. Achieving high reproducibility is essential to ensure rigorous and unbiased experimental design, data acquisition and analysis, thereby facilitating the translation of top-down quantitative proteomics into clinical applications [87]. In contrast to BUP, where standardized analytical workflows have yet to be established over the past decade, TDP remains highly sensitive to variations in sample preparation, LC or CE separation and MS instrumentation [89]. Standardization efforts coordinated by community-driven initiatives facilitate inter-laboratory comparisons aimed at identifying methodological challenges and improving reproducibility [65,90]. For example, a protocol describing commonly used biological buffers, standardized sample preparation procedures, and performance benchmarks was established based on a best-practices and benchmarking study conducted by the Consortium for TDP [65]. In addition to reproducibility and analytical standardization issues, sample throughput is another major limitation to the application of proteoform measurements in clinical studies, which often require analysis of hundreds to thousands of samples to achieve reliable statistical inference. Compared with MALDI-based approaches for proteoforms analysis, ESI-based top-down MS remains limited by relatively low sample throughput [91], highlighting the need for the optimized analytical workflows, particularly in sample preparation and separation techniques. Automation of sample preparation workflows offers a promising avenue to facilitate the clinical translation of top-down proteomics by improving analytical consistency, reproducibility, and overall workflow efficiency. To meet the growing demand for automated online ESI interfaces supporting high-throughput proteoform analysis, the Kelleher group developed the SampleStream platform, which has demonstrated a robust and scalable workflow for the association of proteoforms to phenotypic outcomes at high-throughput [92,93]. Furthermore, while online LC and CE have been widely used in TDP workflows, the rapid separation capability of ion mobility techniques suggests that further advances in front-end ion mobility technologies could enable faster and more robust protein separations [94].

Although significant progress has been achieved in LC- or CE-based separation techniques and MS instrumentation, computational and bioinformatics challenges remain a limitation to the application of TDP. In contrast to peptide-centric BUP, TDP generates highly complex MS spectra containing overlapping charge states, isotopic distributions, and fragment ions derived from multiple proteoforms, complicating spectral interpretation. Therefore, reliable deconvolution of multiple proteoform spectra is essential for converting raw mass spectra into monoisotopic masses and charge assignments. Several deconvolution algorithms have been widely used for proteoform identification, including TopFD and FLASHDeconv [90,95,96]. However, inconsistencies in precursor mass and charge assignments among these algorithms contribute to variability in identification assignments [90]. In addition, proteoform identification is computationally challenging due to the enormous combinatorial diversity arising from genetic variation, alternative splicing, and PTMs [97]. Traditional protein sequence databases are often insufficient to represent this combinatorial complexity, leading to expanded database search spaces and increased false discovery rates during proteoform identification. Several alternative identification strategies have been developed, including spectral alignment approaches that enable searches for unexpected PTMs [98], and hybrid database–spectral library strategies that combine protein sequence database and spectral library searches to improve identification success rates and sensitivity [99]. The integration of machine learning and deep neural networks has also significantly improved proteoform identification and characterization through accurate spectral prediction and proteoform spectrum match rescoring [100,101]. Finally, database construction for TDP requires fundamentally different design strategies compared with peptide-centric BUP. Recently, community-driven initiatives have been proposed to generate a definitive reference set of the proteoforms produced from the genome [32,102].

## 3. Aging and Age-Related Diseases

### 3.1. Aging

Aging is one of the most complex biological processes, which leads to a gradual decline in function of organs, tissues and cells, and significant increases in the risks of many age-associated diseases, including cancer, neurodegenerative disorders, and cardiovascular diseases. Thus, understanding the molecular mechanism underlying biological aging is pivotal for identifying interventions and clinical treatments for aging-related diseases [25]. Recently, the Ohtani group have investigated gene expression changes at the protein level using a bottom-up proteomics approach across tissues collected from adult, middle-aged, old, and geriatric mice [103]. However, this approach has intrinsic limitations for identifying and quantifying intact proteoforms with coding polymorphisms, alternative splicing, and PTMs. Therefore, analysis of intact proteins by top-down MS has recently emerged as a powerful tool for better understanding of the molecular mechanisms in aging biology [104,105,106,107,108] (Table 1). Cellular senescence is a hallmark of biological aging defined as a stable state of cell-cycle arrest that occurs in response to various intrinsic and extrinsic stresses [109]. Its activities, linked to the activation of a senescence-associated secretory phenotype (SASP), contribute to organismal aging and chronic age-associated diseases [110]. The Kelleher group have recently investigated proteoform-level changes across HRASG12V-induced senescence in human fibroblasts using a TDP approach [107]. This study identified multiple forms of the proteins associated with the SASP or aging biology, such as distinct proteoforms of HMGA1, GROα and interleukin-8. TDP analysis revealed that the molecular complexity of IL8 proteoforms with a cysteine PTM, S-sulfhydration or S-sulfinic acid, in senescence which have not been previously identified on IL8. S-sulfhydration of NF-κB has been shown to enhance its anti-apoptotic function by promoting DNA binding and transcriptional activation, while H_2_S has recently emerged as a regulator of paracrine senescence [111]. More recently, the Basisty group also have applied quantitative top-down MS for comprehensive characterization and quantitation of the secreted proteoforms related to the SASP in senescent human cells [108]. This study identified a total of 518 proteoforms from 152 unique proteins some of which exhibited statistically significant differences in abundance among the senescent, quiescent and proliferating cells. Notably, HMGA2 proteoforms with N-terminal acetylation and multiple phosphorylation events are differentially expressed, indicating that they may serve as potential biomarkers for cellular senescence. Indeed, stress-activated kinases such as MAPKs and AKT are implicated in regulating these modifications and shaping SASP composition. Through phosphorylation-mediated control of secretion, stability, and activity, SASP components influence inflammation, tissue remodeling, and immune surveillance [112,113].

### 3.2. Cancers

Many diseases, including cancer, neurodegenerative disorders, and cardiovascular diseases, have been associated with changes that result in proteoform alterations through diverse biological processes such as coding polymorphisms, alternative splicing, and PTMs. In cancer biology, numerous studies have revealed the critical roles of protein composition and drift in cancer development and progression, and therapeutic resistance. Therefore, the discovery and characterization of cancer-associated protein biomarkers at the proteoform level has become areas of significant clinical and translational interest, especially in breast, colorectal, ovarian, and lung cancers [19,114,115,116,117,118,119,120] (Table 2). For example, the Sun group have recently reported the first TDP analysis comparing metastatic and nonmetastatic colorectal cancer (CRC) cells [19]. In this study, 111 proteoforms were identified, including TP53 and DNA mismatch repair protein Msh6 (MSH6) variants containing single amino acid variants (SAAVs) of 82 genes from the two cell lines. Notably, TP53 and MSH6 proteoforms harboring SAAVs were strongly associated with an increased CRC metastasis according to the nucleic acid data [121]. Furthermore, quantitative TDP analysis revealed 460 proteoforms corresponding to 248 proteins that exhibited statistically significant differences in abundance between the two cell lines. Among these, several differentially expressed proteoforms of Calmodulin1 (CALM1), Jupiter microtubule associated homolog 1 [JPT1 (HN1)], and Epithelial cell adhesion molecule (EPCAM) were highlighted, all of which have been previously reported to be involved in cancer metastasis. CALM-dependent signaling pathways play key roles in cancer metastasis [122]. JPT1 (HN1) promotes metastatic progression through activation of the nuclear factor κB signaling pathway [123]. The epithelial cell adhesion molecule (EPCAM), a cell-surface glycoprotein, is a critical regulator of tumor biology, particularly in colorectal cancer (CRC), and has emerged as a promising therapeutic target [124]. Most recently, the Yates III group presented a native top-down proteomics (nTDP) strategy to identify protein complexes in breast cancer cells (MCF-7) and in an epidermal growth factor receptor (EGFR)-overexpressed cells (MCF-7-EGFR), a model of resistance to estrogen receptor-α (ER-α) targeted therapies [114]. This study identified 104 distinct complexoforms corresponding to 17 protein complexes. Among these, novel heterodimeric assemblies of triosephosphate isomerase (TPI), heterotrimeric assemblies of macrophage migration inhibitory factor (MIF), homodimeric assemblies of superoxide dismutase [Cu-Zn] (SOD1), and heterodimeric assemblies of nuclear transport factor 2 (NUFT2) were identified as key molecular features of the breast cancer proteome. Interestingly, a significant decrease in the endogenous NUTF2 dimeric complexoforms were observed in MCF-7-EGFR when compared to MCF-7 cells, revealing that EGFR induces the dissociation of NUTF2 dimeric assemblies that modulate ER-α activity. Furthermore, the study demonstrated that EGFR signaling alters the K4 and K55 PTM code to modulate ER signaling and cell growth. In this study, NUTF2 suppressed breast cancer growth via ER-independent mechanisms, while also influencing ER DNA binding and associated protein interaction networks. Conversely, tamoxifen altered the genomic localization of NUTF2. Rather than direct molecular interactions, the study proposed that NUTF2 and ER independently modulate chromatin states, thereby shaping cellular responses to each other’s signaling pathways.

### 3.3. Neurodegenerative Diseases

Neurodegenerative proteinopathies, including Alzheimer’s disease, Parkinson’s disease, and Amyotrophic lateral sclerosis (ALS), are a group of diseases associated with the abnormal misfolding, aggregation, and deposition of specific proteins in various tissues. These pathogenic protein assemblies such as *β*-sheet-rich amyloid structures, which disrupt cellular function and ultimately drive progressive neurodegeneration. Heterogeneity in PTMs of amyloidogenic proteins results in the generation of multiple proteoforms, each of which may modulate disease pathogenesis [125]. Therefore, investigating neurodegenerative diseases at the proteoform level, rather than at the proteome level, is essential for elucidating the molecular complexity underlying these neurodegenerative diseases [126,127,128,129,130,131,132,133,134] (Table 3). For example, ALS is a progressive neurodegenerative disorder characterized by degeneration of motor neurons in the central nervous system. The Cooper group employed native ambient MSI to investigate the spatial distributions of intact superoxide dismutase 1 (SOD1) protein-metal complexes in SOD1^G93A^ transgenic mice, a well-established ALS model [126]. Intact protein analysis revealed that metal-deficient dimeric SOD1^G93A^ complexes are significantly more abundant in motor-associated central nervous system regions implicated in motor neuron degeneration whereas fully metalated SOD1^G93A^ forms displayed a more uniform distribution throughout the tissue. The study further demonstrated that the dimer-destabilizing PTM, glutathionylation, has limited influence on the spatial distribution of SOD1 dimers. Together, these findings provide important molecular insights into the localized abundance of SOD1 demetalation states and their potential roles in ALS pathogenesis. In relation to this study, recent investigations of sporadic ALS in humans further indicate that altered copper bioavailability may contribute to disease progression by broadly impairing cuproenzyme function [135]. Most recently, the Petyuk group have investigated associations between proteoforms and clinicopathological phenotypes of Alzheimer’s disease (AD) using a quantitative TDP approach across 103 human brain tissues [127]. In this study, a total of 1213 proteins and 11,782 distinct proteoforms were identified, of which 154 proteoforms showed significant associations with a clinical and neuropathological trait. Interestingly, C-terminal truncated and N-terminal truncated forms of amyloid beta (Aβ) were differentially associated with cerebral amyloid angiopathy and cognitive function, respectively. Furthermore, this analysis revealed that six proteoforms of VGF, also known as secretogranin VII, had associations with cognitive decline independent of pathology. TLQP-21 modulates microglial function, while TLQP-62 mitigates Aβ plaque burden and disease-associated microglial activation, supporting a role for VGF-derived peptides in neuroimmune regulation [136,137]. Consistently, several VGF peptides show reduced abundance in the brain, cerebrospinal fluid, and plasma in Alzheimer’s and Parkinson’s diseases [138].

### 3.4. Cardiovascular Diseases

Cardiovascular disease (CVD) has been the leading cause of morbidity and mortality worldwide. As the proportion of older adults continues to increase worldwide, population aging has emerged as a major risk factor for CVD [139]. Protein biomarkers such as cardiac troponins, creatine kinase–MB, brain natriuretic peptide, and C-reactive protein, are critical for the diagnosis, prognosis of CVDs [140]. In addition, specific apolipoproteins also play a critical role in regulating lipid transport and the associated metabolic processes and serve as clinically relevant biomarkers that predict cardiovascular risk, including apolipoprotein A-I (ApoA-I) and apolipoprotein C-III (ApoC-III). For example, ApoA-I, major protein component of high-density lipoprotein (HDL) particles, facilitates reverse cholesterol transport [141]. Higher levels of serum ApoA-I are associated with higher HDL cholesterol (HDL-C) and increased HDL-mediated cholesterol efflux [142]. In addition to ApoA-I, ApoC-III, a small apolipoprotein secreted from the liver and small intestine, is involved in triglyceride metabolism. Higher level of serum ApoC-III is linked to higher triglycerides, increased low-density lipoprotein cholesterol (LDL-C), and lower HDL-C cholesterol [143]. Both ApoA-I and ApoC-III exist in multiple proteoforms [93,144]. Because alternative splicing and PTMs regulate the function of these proteins, it is essential to study CVD at the proteoform level to gain new insights into the molecular mechanisms underlying CVDs [144,145,146,147,148,149,150] (Table 4). Among CVDs, hypertrophic cardiomyopathy (HCM) is recognized as one of the most common inherited cardiac disorders. Recently, HCM has been identified in older adults presenting with common cardiovascular risk factors, and old age in individuals with HCM is regarded a negative risk marker for sudden cardiac death [151,152]. The genetic cause of HCM has been associated with mutations in genes that encode sarcomeric proteins. Nevertheless, the ability to predict heart disease events based on specific mutations in HCM patients is still limited. The Ge group employed TDP for comprehensive characterization of sarcomeric proteoforms across tissues from HCM patients [145]. Notably, the analysis demonstrated consistent alterations in phosphorylated proteoforms of sarcomeric proteins, including cardiac troponin I, cardiac troponin T, enigma homolog isoform 2, tropomyosin isoforms, and myosin light chain 2, between donors and HCM patients despite different HCM-causing mutations. Notably, sarcomeric proteoforms were consistently remodeled in the myocardium of HCM patients irrespective of causal mutations or confounding influences. Decreased phosphorylation of cTnI and ENH2 exhibited a strong linear correlation in HCM compared with donor tissues, supporting dysregulation of PKA-mediated signaling and functional PTM cross-talk between myofilament and Z-disk components. These results imply that diverse mutations in sarcomeric genes such as *MYH7* and *MYBPC3* may ultimately converge on common pathogenic mechanisms, producing shared proteoform signatures associated with comparable obstructive HCM phenotypes.

## 4. Conclusions

MS-based proteomics is a powerful technology for characterizing entire proteins from complex biological systems such as cells, tissues, and organisms. The BUP and TDP techniques show complementary performances. In contrast to BUP measuring peptides produced from proteins by proteolytic digestion, TDP allows for directly measuring proteins without proteolysis, thus providing proteoform-resolved information on coding polymorphisms, alternative splicing events and PTMs on the same molecule present in vivo. Although there still remains many technical challenges of TDP for complete characterization of large proteins and protein complexes, continued advances in separation techniques and MS instrumentation will help to develop a deep sense of the functions of genetic variation, alternative splicing and PTMs. With improving technology, TDP studies are pivotal for better understanding their roles in complex biological and various diseases events. While recent studies introduced in this review have demonstrated the potential of TDP to characterize and quantify proteoforms associated with aging and age-related diseases, several reported biomarker candidates originate from limited experimental systems, small cohort sizes, highlighting the need for sufficient validation across independent cohorts, analytical platforms, or biological contexts. Small-scale discovery studies remain valuable for hypothesis generation, particularly given the technical complexity and relatively low throughput associated with TDP workflows. However, limited sample numbers may increase susceptibility to statistical overfitting, cohort-specific biases, and confounding variables such as age heterogeneity, comorbidities, medication status, and sample processing differences. These factors can significantly influence proteoform distributions and PTM patterns, thereby challenging the reproducibility of reported biomarker signatures. Thus, candidate biomarkers identified in single-laboratory studies should be regarded as preliminary until confirmed through orthogonal validation approaches, including immunoassays recognizing specific proteoforms, or independent replication using alternative instrumentation.

Despite significant advances in TDP over the past decade, several technological challenges remain. Future developments are expected to drive progress in sample preparation, MS instrumentation, computational analysis, and translational integration with clinical research. First, emerging areas, including spatial and single cell TDP technologies, is more promising for research on aging and age-related diseases. Cellular heterogeneity increases with age, and tissue-level measurements may limit the resolution of key cell type-specific proteoform changes. Spatial and single cell resolved technologies could uncover previously uncharacterized molecular signatures associated with aging and age-related diseases, including neurodegeneration, cardiovascular disorders, and cancer. Second, continued improvements in MS instrumentation such as individual ion MS, which is an Orbitrap-based MS technique known as charge detection MS, are anticipated to improves measurement of highly complicated mixtures of proteoforms and their complexes, particularly those with high molecular weight or low abundance. Enhancing sensitivity and dynamic range will be particularly important for aging-related studies, changes in low-abundance proteoforms may serve as early-stage indicators of biological decline or disease onset. Finally, computational and bioinformatic advancements represent a major frontier for TDP. The identification and quantification of proteoforms and their complexes remain computationally demanding due to spectral complexity and incomplete proteoform databases. Machine learning and artificial intelligence-driven algorithms are expected to accelerate spectral interpretation, enhance proteoform identification confidence, and enable large-cohort analyses.

## Figures and Tables

**Table 1:** Aging-associated proteoform alterations revealed by TDP.

Sample Type	Protein	Proteoform Alteration	Ref.
Skeletal muscle	Myosin regulatory light chain (RLC)	Age-dependent decrease in RLC phosphorylation (Ser14/Ser15).	[104]
Skeletal muscle	Cypher	In GAS muscle, phosphorylation of Cypher isoforms (Cypher4s and Cypher2s) decreases with age.	[105]
Heart tissue	NADH dehydrogenase [ubiquinone] iron-sulfur protein 6 (NDUS6) Cytochrome c oxidase subunit 6B1 (CX6B1) NADH dehydrogenase [ubiquinone] flavoprotein 3 (NDUV3) Glutaredoxin-related protein 5 (GLRX5)	NDUS6, CX6B1, NDUV3 and GLRX5 were reduced in old hearts compared to young hearts.	[106]
Heart tissue	Mitochondrial import inner membrane translocase subunit Tim8 A (Tim8A) Electron transfer flavoprotein subunit beta (ETFB) Malate dehydrogenase (MDHM)	Tim8A, ETFB and MDHM were increased in old hearts compared to young hearts.	[106]
Heart tissue	NADH dehydrogenase ubiquinone 1 alpha subcomplex subunit 2 (NDUA2)	In aging hearts, the abundance of NDUA2 proteoforms decreases, particularly the oxidized and succinylated form, while its relative proportion compared to the N-terminally acetylated form increases.	[106]
Cell	Senescence-associated secretory phenotype (SASP) proteins	Proteoform-level changes including IL-8 N-terminal cleavage and novel cysteine S-sulfhydration/sulfinic acid modification, HMGA1 multi-phosphorylation and Arg25 methylation, proteoform variation in CXCL5 and GROα, downregulation of caspase-3 and caspase-7 (apoptosis escape), and upregulation of caspase-4, EIF2AK2, and PRKRA.	[107]
Cell	High mobility group AT-hook 2 (HMGA2)	HMGA2 proteoforms with N-terminal acetylation and multiple phosphorylation states (di-, tri-, tetra-phosphorylated) were elevated in senescent cells.	[108]

**Table 2:** Cancer-associated proteoform alterations revealed by TDP.

Disease	Sample Type	Protein	Proteoform Alteration	Ref.
Colorectal cancer	Cell	Tumor protein p53 (TP53) and MutS homolog 6 (MSH6) proteoforms containing SAAVs	TP53 and MSH6 proteoforms with SAAVs were strongly associated with increased metastatic potential in colorectal cancer.	[19]
Breast cancer	Cell	Nuclear transport factor 2 (NUTF2)	EGFR signaling reduced endogenous NUTF2 dimeric complexoforms and altered K4 and K55 PTMs, modulating ER-α activity.	[114]
Breast cancer	Tissue	Heterogeneous nuclear ribonucleoproteins A2/B1 (HNRNP A2/B1)	The C-terminal proteoform of HNRNP A2/B1 (*m*/*z* 3357) is upregulated in breast cancer tissues and correlates with estrogen receptor expression.	[115]
Colorectal cancer	Tissue	Reticulocalbin (RCN) Calumenin (CALU)	Increased expression levels of reticulocalbin and calumenin.	[116]
Colorectal cancer	Cell	Histone	All nine histone H4 proteoforms and two histone H2A proteoforms (with N-terminal acetylation and phosphorylation) are upregulated in metastatic colorectal cancer cells.	[117]
Ovarian cancer	Tissue	Cysteine-rich intestinal protein 1 (CRIP1)	Methylated CRIP1 proteoforms show differential spatial localization, being enriched in tumor regions.	[118]
Lung cancer	Serum	Neuron Specific Enolase Gamma (NSEγ)	NSEγ showed a +42 Da shift consistent with single acetylation, and only the acetylated proteoform was detected in serum.	[119]
Colorectal cancer	Tissue	Kirsten rat sarcoma viral oncogene homolog 4b (KRAS4b)	KRAS4b proteoforms exhibited cysteine 118 nitrosylation and variable levels of C-terminal carboxymethylation, indicating mutation-specific PTM heterogeneity.	[120]

**Table 3:** Neurodegenerative disease-associated proteoform alterations revealed by TDP.

Disease	Sample Type	Protein	Proteoform Alteration	Ref.
Amyotrophic lateral sclerosis	Tissue	Superoxide Dismutase 1^G93A^ (SOD1^G93A^)	Metal-deficient SOD1^G93A^ proteoforms accumulated in motor-associated central nervous system regions and correlated with motor neuron loss.	[126]
Alzheimer’s disease	Tissue	Amyloid-β (Aβ)	C-terminally truncated Aβ proteoforms distinguished amyloid plaque from CAA pathology, while N-truncated Aβ_x–42_ correlated with cognitive decline.	[127]
Alzheimer’s disease	Tissue	Amyloid-β (Aβ)	N- and C-terminally truncated Aβ proteoforms were abundant in AD brain; canonical Aβ1–40/42 constituted only a minor fraction, highlighting extensive proteoform heterogeneity.	[128]
Alzheimer’s disease	Saliva	Salivary antimicrobial and antioxidant defense proteins	Oxidized and nitrosylated S100A8/A9, glutathionylated Cystatin B, and elevated α-defensins and Histatin-1 reflected oxidative stress and immune activation.	[129]
Alzheimer’s disease	Tissue	Tau Neurogranin Calmodulin-1	Differentially quantified tau, neurogranin, and calmodulin-1 proteoforms were associated with AD-related pathways.	[130]
Parkinson’s disease	Cerebrospinal fluid	Phosphorylated α-synuclein (PS-129)	The ratio of PS-129 to α-synuclein was increased in PD.	[131]
Alzheimer’s disease	Tissue	Tau	Multiple tau isoforms (0N3R, 1N3R, 0N4R, 1N4R) showed extensive PTMs and truncations, and small truncated tau proteoforms (~10 kDa) were elevated in AD brain.	[132]
Parkinson’s disease	Tissue	α-Synuclein (αSyn)	N- and C-terminally truncated α-synuclein proteoforms retaining an intact NAC domain were identified in the human appendix.	[133]
Parkinson’s disease	Recombinant α-synuclein proteoforms	α-Synuclein (αSyn)	O-GlcNAc-modified α-synuclein proteoforms at T72, T75, T81, and S87 were resolved by top-down MS, revealing positional glycoform heterogeneity.	[134]

**Table 4:** Cardiovascular disease-associated proteoform alterations revealed by TDP.

Disease	Sample Type	Protein	Proteoform Alteration	Ref.
Cardiovascular disease	Serum	Apolipoprotein A-I (ApoA-I)	Multiple proteoforms (truncation, oxidation, PTMs) are associated with HDL-E capacity.	[144]
Hypertrophic cardiomyopathy (HCM)	Heart tissue	Sarcomeric proteins	cTnT phosphorylation significantly increased, while the phosphorylation of Tpm isoforms, MLC-2, and ENH2 consistently decreased.	[145]
Cardiovascular disease	Serum	Apolipoprotein C-III (ApoC-III)	Altered proteoforms with O-glycosylation and sialylation features (ApoC-III_0_, ApoC-III_1_, ApoC-III_2_).	[146]
Cardiovascular disease	Heart tissue	Cardiac troponin I (cTnI)	Cardiovascular disease is associated with reduced phosphorylation of cTnI.	[148]
Cardiovascular disease	Heart tissue	Muscle LIM protein (MLP) Calsarcin-1 (Cal-1)	Increased phosphorylation of MLP and Cal-1 in diseased hearts compared with controls.	[149]
Cardiovascular disease	Heart tissue	α-Cardiac actin (αCAA) α-Skeletal actin (αSKA)	Altered actin isoform composition in heart tissue from cardiac disease patients, with increased αSKA levels.	[150]

## Data Availability

No new data were created or analyzed in this study. Data sharing is not applicable to this article.
